# Bayesian uncertainty estimation for detection of long-tailed and unseen conditions in medical images

**DOI:** 10.1117/1.JMI.10.5.054501

**Published:** 2023-10-09

**Authors:** Mina Rezaei, Janne J. Näppi, Bernd Bischl, Hiroyuki Yoshida

**Affiliations:** a LMU Munich, Department of Statistics, Munich, Germany; b Munich Center for Machine Learning, Munich, Germany; c Massachusetts General Hospital, Harvard Medical School, 3D Imaging Research, Department of Radiology, Boston, Massachusetts, United States

**Keywords:** deep Bayesian modeling, uncertainty estimation, long-tailed distribution learning, out-of-distribution learning

## Abstract

**Purpose:**

Deep supervised learning provides an effective approach for developing robust models for various computer-aided diagnosis tasks. However, there is often an underlying assumption that the frequencies of the samples between the different classes of the training dataset are either similar or balanced. In real-world medical data, the samples of positive classes often occur too infrequently to satisfy this assumption. Thus, there is an unmet need for deep-learning systems that can automatically identify and adapt to the real-world conditions of imbalanced data.

**Approach:**

We propose a deep Bayesian ensemble learning framework to address the representation learning problem of long-tailed and out-of-distribution (OOD) samples when training from medical images. By estimating the relative uncertainties of the input data, our framework can adapt to imbalanced data for learning generalizable classifiers. We trained and tested our framework on four public medical imaging datasets with various imbalance ratios and imaging modalities across three different learning tasks: semantic medical image segmentation, OOD detection, and in-domain generalization. We compared the performance of our framework with those of state-of-the-art comparator methods.

**Results:**

Our proposed framework outperformed the comparator models significantly across all performance metrics (pairwise t-test: p<0.01) in the semantic segmentation of high-resolution CT and MR images as well as in the detection of OOD samples (p<0.01), thereby showing significant improvement in handling the associated long-tailed data distribution. The results of the in-domain generalization also indicated that our framework can enhance the prediction of retinal glaucoma, contributing to clinical decision-making processes.

**Conclusions:**

Training of the proposed deep Bayesian ensemble learning framework with dynamic Monte-Carlo dropout and a combination of losses yielded the best generalization to unseen samples from imbalanced medical imaging datasets across different learning tasks.

## 1 Introduction

**Fig. 1:**
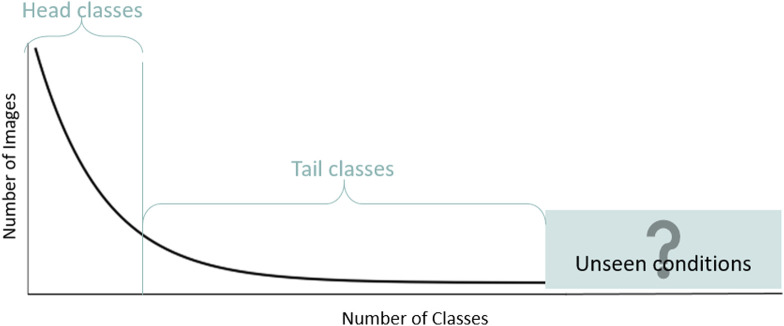
A model should be trained with long-tailed training data such that it can generalize to unseen-conditions test data.

Real-world medical imaging data, such as those used for semantic segmentation of multiple organs and lesions on CT images, tend to have inherently long-tailed distributions with a few standard classes and many rare (tail) classes. The low number of training samples in the tail classes makes it challenging to learn optimal classification boundaries in the feature space. Under such conditions, a deep learning model needs to classify between a few high-frequency and many low-frequency categories while also being able to generalize based upon instances of previously infrequently occurring varieties (Fig. 1). We formulate the task of detecting the infrequent samples from long-tailed categories as an out-of-distribution (OOD) detection problem.

Possible solutions for handling the long-tailed distribution include modification of the data distribution^1^ and adjustment of reasonable costs to reweight class errors.^2^^,^^3^ However, the existing data-level approaches are prone to overfitting, whereas existing cost-sensitive learning methods require a careful choice of weights. As discussed by Fort et al.,^4^ these approaches have not performed well in rare conditions. In the literature, OOD detection usually refers to solving domain shift or distribution shift problems, and the difficulty of this task depends on how semantically close the outliers are to the inlier classes. Recently, Winkens et al.^5^ described experiments between detecting challenging near-OOD tasks and easy far-OOD tasks.

There has also been considerable attention on modeling uncertainty in a trustworthy manner in machine-learning and deep learning deployments in healthcare. Predictive uncertainty estimation^6^ plays an essential role in reducing uncertainties during both optimization and decision-making. Bayesian approximation and ensemble learning models are two of the most successful techniques for estimating uncertainties. This paper introduces a new direction toward the representation learning of long-tailed and OOD data. Motivated by the recent progress in uncertainty modeling, we propose an uncertainty-aware estimation framework by quantifying uncertainties associated with the predicted class probabilities by use of a generative multi-discriminative framework to address the more challenging problem of detecting near-OOD tasks or infrequent samples from the long-tailed data distribution. Our method is based on the observation that rare classes have a higher uncertainty and wider confidence intervals in the prediction space than do the more frequently occurring classes. Therefore, by incorporating the uncertainty estimates, we can expand the decision boundaries to the less frequent classes to help the classifier’s generalization toward unseen conditions. Specifically, we propose to incorporate this uncertainty in identifying reliable examples using an ensemble of networks and by assigning the class labels based on a consensus of high-confidence predictions.

The proposed framework consists of a deep generator and multiple deep discriminator networks. In the application of semantic segmentation of medical images, the generative probabilistic model builds the model based on prior domain knowledge of the appearance and spatial distribution of the different image patch types. In contrast, the discriminative model directly learns the relationship between the local features of images and the true label distribution.

The key contributions of this paper can be summarized as follows. 1. We introduce a Bayesian ensemble generative adversarial network (GAN), which is a new type of adversarial framework for learning the representation of the long-tailed data distribution and for detecting OOD samples. 2. We develop and train our framework by incorporating different uncertainty conditions. 3. We apply a principled approach to integrate Bayesian uncertainty estimates for learning the class imbalance. 4. We demonstrate that the training of the proposed network with a dynamic Monte-Carlo (MC) dropout and a combination of losses yields a better generalization of the learned classifier to unseen samples than without these methods. 5. We demonstrate and evaluate the application of our proposed model on four public medical imaging datasets for three tasks considering different image modalities.

## 2 Related Work

This section describes related work in the area of deep learning approaches for quantifying uncertainties and for learning representations of imbalanced data and OOD samples.

### 2.1 Deep Learning Methods for Quantifying Uncertainties

In the medical literature, the Bayesian approach and an ensemble of deep learning networks are the most widely used methods for quantifying uncertainties.^7^^,^^8^ Abdar et al.^9^ applied an MC dropout method called ensemble MC dropout for quantifying uncertainties in skin cancer detection. Nair et al.^10^ developed a 3D convolutional neural network model to estimate multiple uncertainties at the voxel level for the task of lesion detection and segmentation in brain images. PU-Net^11^ combined the conditional variational autoencoder^12^ and a U-Net^13^ to capture uncertainties by use of a downsampled axis-aligned Gaussian prior that was updated through the Kullback–Leibler divergence of the posterior.

In this paper, we propose to incorporate uncertainty in identifying reliable examples using an ensemble of networks and by assigning the class labels based on a consensus of high-confidence predictions.

### 2.2 Deep Learning Models for Detecting OOD Samples

Hendrycks and Gimpel^14^ used the maximum predicted class probability by a deep-learning model as a confidence score that a sample is OOD. Later, an OOD detector for neural networks (ODIN) model^15^ was developed to extend this framework by applying an adversarial perturbation to the input image and by adding a temperature scaling before softmax to increase the difference between the prediction probabilities of in-distribution and OOD samples. Generalized ODIN^16^ extended these previous studies by defining an additional output that indicated whether the input sample belongs to the training distribution or OOD.

In this paper, we address the detection of near-OOD tasks or infrequent samples from the long-tailed data distribution by formulating the problem with a new uncertainty-aware deep ensemble framework.

### 2.3 Handling Imbalanced Data with GANs

We can divide the current approaches for handling class imbalance problems using GANs into two types of methods: data level and algorithm level. At the data level, GANs are used widely for internal bias correction by generating or synthesizing training data for the minority classes.^17^^,^^18^ At the algorithm level, conditional GANs are used with modification of the training loss^19^^,^^20^ or ensemble learning.^21^ Most of the GAN-based ensemble techniques modify the network architecture by training generative multi-discriminative networks,^22^^,^^23^ multi-generative discriminative networks,^24^ or a cascade of GANs.^25^^,^^26^ For a comprehensive literature survey of GAN-related algorithms, we refer the reader to Ref. 27.

## 3 Methods

We propose to develop a deep Bayesian ensemble GAN that learns a balanced representation of the input data. Our method is based on the observation that tail classes have a higher uncertainty and wider confidence intervals in the prediction space than the other classes. Therefore, we can utilize a quantification of the uncertainties to expand the decision boundaries toward less frequent classes. In this section, we first describe our motivation for using Bayesian uncertainty estimation and ensemble models. We will then introduce our new loss functions, the ensemble architecture, and the details of the optimization.

### 3.1 Bayesian Uncertainty Estimation

In addition to output predictions, Bayesian models can also estimate uncertainty. Given an input, the uncertainty approximations correspond to the confidence level for each outcome predicted by the model. Because the confidence level of predictions is directly associated with class representation in the training set, samples from the tail classes of the training set have higher uncertainty. In contrast, the classifier’s confidence levels are low. Therefore, we developed a dynamic MC dropout to estimate the Bayesian uncertainty. A dropout-based deep network provides an approximation to the Gaussian process^28^ that constructs the prior distribution. This distribution is updated conditionally on the observations, i.e., all the functions consistent with the labels are retained. In the testing phase, the output is obtained from each of the functions, and the expectation is computed to generate the final prediction. The variance of these outputs gives an uncertainty estimate. In the following sections, we will first provide an overview of the dropout method and describe the uncertainty computation with our proposed dynamic MC dropout method.

### 3.2 Dropout

Srivastava et al.^29^ proposed the dropout method as a regularization term for deep neural networks. During training, a subnetwork is sampled from the whole network by randomly dropping a set of neurons, and each neuron is activated with a fixed probability p. The weights are modified at each neuron by injecting Gaussian noise during training time.

### 3.3 Dynamic Dropout

Deep neural networks include d layers and parameters that can be modeled as a function $f_{\theta }$, where $\theta =\{\theta_{1},\ldots ,\theta_{d}\}$ denote the parameters of each layer. By applying a Gaussian distribution δ∼N(1,σ), we can obtain N samples corresponding to the different network configurations θ^ that form an ensemble network O={θ^i:i∈[1,N]}, where θ^i=θ·δ. Given a randomly sampled mini-batch of N input images $\left\{x_{1},x_{2},\ldots ,x_{N}\right\}$, the model configurations are applied to predict a set of outputs {y^}. The aggregate output is computed by the MC estimate by the first moment Eq(y|x)[y]:y≈1NΣi=1Ny^(x,θ^i). Here q indicates an output distribution that approximates the intractable posterior distribution of the deep Gaussian process. The uncertainty is estimated by the second moment $V_{q(y|x)}[y]$ through the MC: u≈τ−1IC+1NΣi=1Ny^Ty^−Eq(y|x)[y]TEq(y|x)[y], where $I_{C}$ is an identity matrix with C indicating the number of classes, and τ indicates the normalized class frequencies.

### 3.4 Ensemble GAN

We use a deep ensemble GAN with a modified dynamic dropout as the ensemble network to obtain the Bayesian uncertainty estimates. We train an ensemble of the generator and multi-discriminators to boost the predictive performance and use adversarial training to improve the algorithm’s robustness. Our framework comprises a single generator G and a set of multi-discriminator variants. The multi-discriminator variants are used to improve the approximation of $\max\;V(G,D_{k})$ by providing an enhanced critique to the generator. Here the generator learns from the feedback, aggregated over the multiple discriminators by $\sum_{k=1}^{K}V(G,D_{k})$, which forces the generator G to learn and minimize the prediction error of semantic segmentation through the ensemble of discriminators. This ultimately encourages G to produce conditional samples with a minimum error since G needs to fool all the different possible discriminators. Heterogeneity in the ensemble is achieved by the feedback of each D (average, maximum, or sum) with a specified probability at the end of every batch. Therefore, G will only consider the losses of the remaining discriminators in the ensemble when updating its parameters at each iteration.

### 3.5 Objective Function

We implemented the proposed Bayesian ensemble GAN with a cohort of four networks. Here a single generator attempts to minimize the segmentation error based on an ensemble of k other losses. The generator takes a random vector z and medical images x as input. In contrast, three discriminators attempt to minimize the error of predicting the segmentation masks produced by the generator through the multiple losses. Here for a fixed G, function F will receive either sum, average, or maximum of k different discriminator losses to the generator through the objective of $\min_{G}\;\max_{D_{k}}\;F(V(D_{1},G),V(D_{2},G),\ldots ,V(D_{k},G)))$, which can be formulated as

$$\underset{G}{\min}\;\underset{D_{k}}{\max}\;V(D_{k},G)=E_{x,y∼p(x,y)}[\log D_{k}(x,y)]+\lambda_{k}E_{z∼p(z),y∼p(y)}[\log(1-D_{k}(G(z,y),y))].$$ (1)

## 4 Experiments

### 4.1 Dataset

We evaluated the performance of our Bayesian ensemble GAN based on clinical patient data from the following four publicly available challenge datasets.

#### 4.1.1 Liver tumor segmentation

The liver tumor segmentation (LiTS) benchmark^30^ of the Medical Image Computing Computer Assisted Intervention (MICCAI) 2017 conference contains 130 training and 70 test CT cases, where the patients have different types of liver cancers.^31^ The challenge was to perform a simultaneous semantic segmentation of a large liver with a 1:400 imbalanced class ratio of pixels representing the liver and the surrounding tissue together with an abnormal target region, with a 1:1400 imbalanced class ratio between pixels representing the abnormal and normal tissue.

#### 4.1.2 Combined healthy abdominal organ segmentation

The combined healthy abdominal organ segmentation (CHAOS)^32^ of the IEEE International Symposium on Biomedical Imaging 2019 conference consists of abdominal CT and MR images, where each image slice has been manually segmented by expert radiologists.^33^ Specifically, it includes 20 MR and 20 CT abdominal images with five segmentation labels for the liver, spleen, left kidney, right kidney, and background. We trained our model on a total of 16,266 2D images with 256×256  pixels and tested on 1793 similarly sized 2D images. Here the imbalance ratios are 1:40, 1:200, 1:400, and 1:400, defined as the number of pixels in the background class to the number of pixels that belong to the regions of the liver, spleen, and left and right kidneys.

#### 4.1.3 Glaucoma detection

Glaucoma detection^34^ is a real-world clinical dataset that includes microscopic retina images from 956 patients with the neuropathic disease glaucoma and from 1401 patients with normal (healthy) retinas. Each input sample is a single red-green-blue image and we resized all images to 128×128. Image augmentation was applied by a combination of crop, horizontal flip, and color jitter. The dataset is imbalanced with a ratio of 1:30.

#### 4.1.4 REFUGE

REFUGE 2020^35^ was a challenge at the MICCAI 2020 conference, focused on retinal glaucoma diagnosis. The dataset comprises 800 microscopic retina images with dimensions of 1411×1411  pixels, collected from various clinics. We used this dataset in tandem with the models trained on the glaucoma detection dataset to evaluate their ability to detect OOD samples using predicted uncertainties. This approach was adopted because the dataset originated from diverse sources and countries.

### 4.2 Implementation and Parameter Configuration

Our framework encompassed a single generator and three discriminators. The generator had a stacked hourglass network architecture^13^ that provides a mechanism for repeated bottom-up and top-down inference, allowing for a re-evaluation of the initial estimates and features across the whole image. The architecture of the discriminator was akin to that of a Markovian discriminator^36^ that is designed to restrict attention to the structure of local image patches. The discriminator losses included the mean absolute error ($\ell_{\mathrm{mae}}$), categorical cross-entropy ($\ell_{\mathrm{cce}}$), and Dice loss ($\ell_{\mathrm{Dice}}$). We used the discriminators that had been pretrained with ImageNet for the initialization of the weights, whereas we trained the generator from scratch using a Gaussian distribution with a standard deviation of 0.001. The learning rate started from 0.0002 with a mini-batch size of 1. We used the Adam optimizer and set $\beta_{1}=0.9$ and $\beta_{2}=0.999$ with a weight decay of 0.0001. Binary cross-entropy was used as the adversarial loss. For all datasets, barring REFUGE 2020 used for OOD detection, we applied a threefold cross-validation to estimate the performance of the trained model, where 80% of the total training dataset was designated for training, and the remaining 20% was employed for validation.

### 4.3 Comparator Methods

We compared our Bayesian ensemble GAN against the following methods within our experimental setting.

#### 4.3.1 Conditional GAN

A standard conditional GAN^37^ was utilized to perform semantic segmentation, using the hyperparameters and settings as outlined by Rezaei et al.^19^

#### 4.3.2 Ensemble GAN

Ensemble GAN is an alternative technique of Bayesian neural network for model uncertainty and a gold standard of epistemic uncertainty. It aims to reduce the variance and to improve the generalization performance of a single deep neural network using the diversity of the ensemble. Each network is trained on the same dataset but with different initial random weights, and the outputs of the networks are combined by averaging. We trained the conditional GAN with random initialization of the weights 10 times and reported the average performance.

#### 4.3.3 U-Net

We used the U-Net^13^ with the same configuration as described by Christ et al.^30^

#### 4.3.4 MC Dropout

We used dropout^28^ as a regularizer to quantify the uncertainty of the prediction.

#### 4.3.5 Masksemble

Masksemble^38^ is an extension of MC dropout with a different approach. Instead of randomly dropping network components during training like in MC dropout, Masksemble uses a fixed number of predefined binary masks that are randomly generated before the training.

#### 4.3.6 BatchEnsemble

BatchEnsemble^39^ leverages low-rank matrices to efficiently construct an ensemble by expanding the layer weights. The method decomposes the weight matrix of a network layer into a matrix that is shared among all members of the ensemble and an individual rank-1 matrix per member. These matrices are then combined using the Hadamard product for expanding the base network into an ensemble.

### 4.4 Task-Specific Evaluations

We conducted experiments across three distinct learning tasks, each with specific objectives. 1. Evaluating the predictive performance of models on in-domain datasets: We conducted experiments on both semantic segmentation and image classification, aiming to obtain high predictive scores alongside low uncertainty scores. 2. Examining the ability of the models to generalize from in-domain to OOD datasets; this was achieved through the OOD detection task, where we targeted high uncertainty scores. 3. Estimating the level of uncertainty demonstrated by the models on OOD datasets, with a specific focus on achieving high uncertainty scores.

We evaluated and compared the performance of the proposed framework and that of the comparator models using several performance metrics. For the semantic segmentation task, these included average symmetric surface distance (ASSD), F1 score, precision, and recall. For the image classification task, we used F1 score, expected calibration error (ECE), and negative log-likelihood.

#### 4.4.1 Semantic segmentation

We evaluated the performance of a model in the task of semantic segmentation using two distinct datasets, namely LiTS and CHAOS. The LiTS dataset was selected to evaluate the model’s capability to simultaneously segment the entire liver and very small lesions in the liver. Furthermore, in the LiTS dataset, lesions with a diameter of 10 mm or more are classified as large, whereas lesions with a diameter of <10  mm are classified as small. Given the imbalanced pixel distribution, the segmentation of the small lesions poses a challenge. On the other hand, our objective of using CHAOS segmentation was to test the model’s ability for multi-organ semantic segmentation.

#### 4.4.2 In-domain generalization

We evaluated the in-domain generalization performance of a model by measuring its ability to make accurate predictions on a test set of the dataset from which the training sets were derived. Specifically, we analyzed the F1 score based on the in-distribution test set.

#### 4.4.3 Out-of-distribution detection

In an OOD detection task, the goal was to identify whether a given input falls within the same distribution as the training data or not. In other words, the aim was to detect whether the input came from the same distribution that the model was trained on, or if it came from an unseen distribution. For this task, we used REFUGE 2020 as an unseen dataset, where the samples were obtained using a different patient population and medical equipment than those of the training dataset.

## 5 Results

In this section, we present a comparative analysis of the performance of our Bayesian ensemble GAN with the comparator models, i.e., the Conditional GAN, U-Net, and the deep ensemble GANs, across the three different learning tasks: semantic segmentation (Sec. 5.1), in-domain generalization (Sec. 5.2), and OOD detection (Sec. 5.3).

### 5.1 Semantic Segmentation

**Fig. 2:**
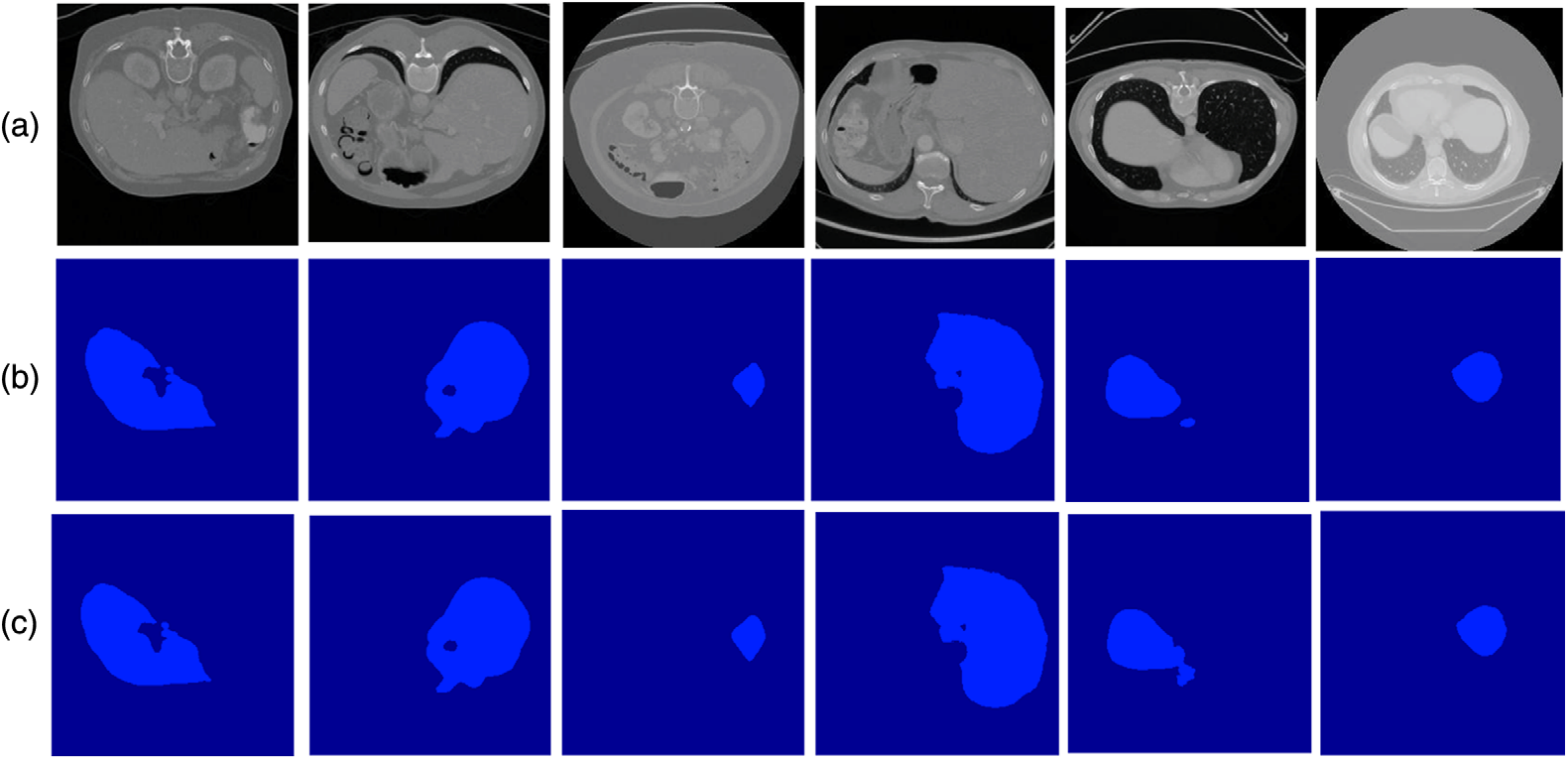
Semantic segmentation results for the LiTS dataset: (a) input image, (b) ground truth image, and (c) prediction by the Bayesian ensemble GAN.

We analyzed the accuracy of the aforementioned models on the imbalanced liver tumor segmentation dataset, characterized by an imbalance of labels between large organs and very small lesions. As shown by our results on the LiTS dataset in Table 1 and Fig. 2, our proposed Bayesian ensemble GAN provides a promising approach for semantic segmentation. Based on the results obtained in Table 1, our approach achieved significantly higher performance than those of the other methods, such as deep ensemble GANs. For each performance metric, a pairwise t-test on the difference in the scores between the Bayesian ensemble GAN and each comparator model showed that the difference in performance was statistically significant (p<0.01).

**Table 1:** Comparative performance of the four models in the semantic segmentation of the LiTS dataset.

	ASSD		F1		Precision		Recall	
	Mean±std	p-value	Mean±std	p-value	Mean±std	p-value	Mean±std	p-value
Bayesian ensemble GAN	6.1±0.2	—	96.2±0.2	—	94.5±0.1	—	91.1±0.2	—
Ensemble GAN	6.2±0.1	0.008	95.3±0.4	0.0021	94.2±0.3	0.0024	89.1±0.6	0.0037
Conditional GAN	10.8±1.2	<0.0001	88.2±2.4	<0.0001	90.1±1.1	<0.0001	79.0±1.0	<0.0001
U-Net	14.7±0.6	<0.0001	82.1±1.1	<0.0001	86.4±0.9	<0.0001	71.1±0.5	<0.0001

Note: The best scores are highlighted in bold.

**Fig. 3:**
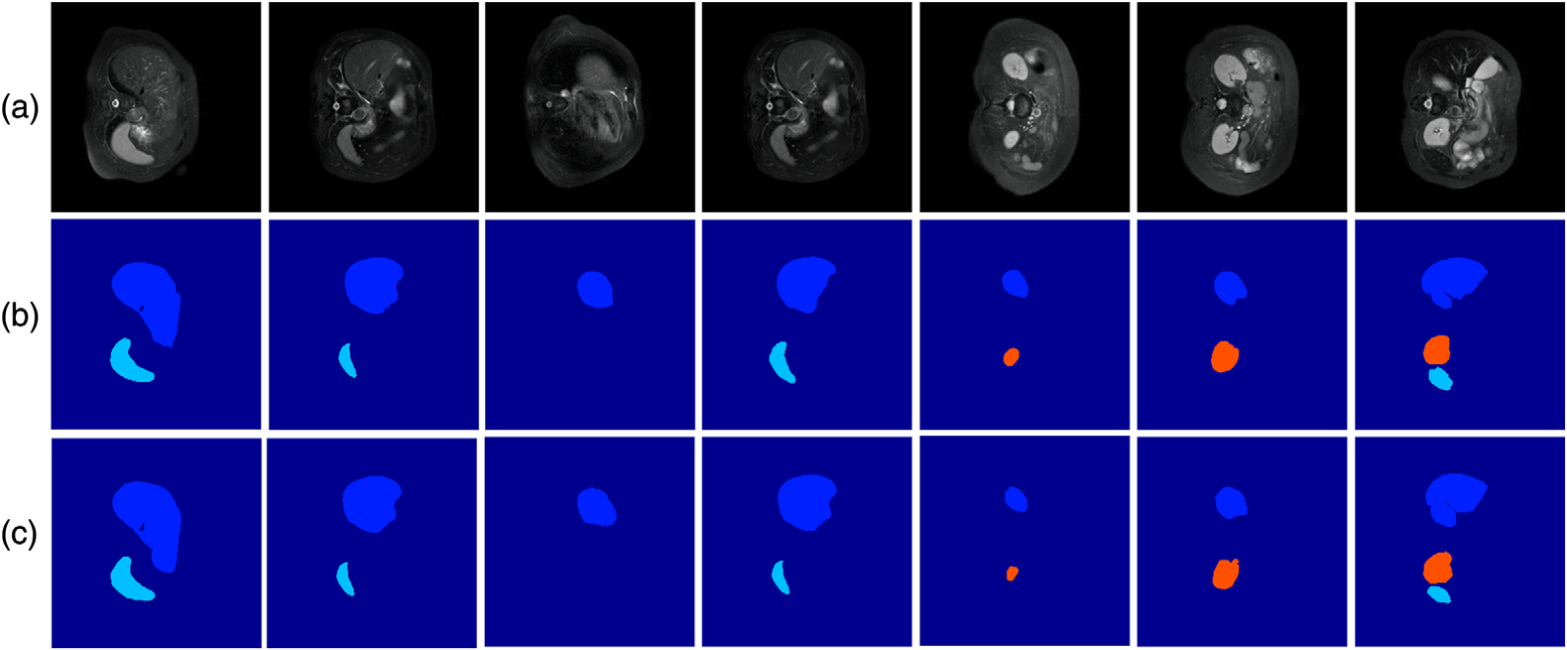
Semantic segmentation results: (a) input image, (b) ground truth image, and (c) prediction by the Bayesian ensemble GAN on the CHAOS dataset for liver, spleen, and kidney, shown in dark blue, light blue, and orange, respectively.

Table 2 and Fig. 3 show the comparative performance of our Bayesian ensemble GAN and the three comparator models on the semantic segmentation of the CHAOS dataset. They show that our method outperforms the three comparator models in predicting semantic segmentation of CHAOS dataset. The quantitative results in Table 2 show that our Bayesian ensemble GAN outperformed the other models in all scores, demonstrating the effectiveness of the proposed method in the semantic segmentation task involving imaging datasets with imbalanced labels. For each performance metric, a pairwise t-test on the difference in the scores between the Bayesian ensemble GAN and each comparator model showed that the difference in performance was statistically significant (p<0.001).

**Table 2:** Comparative performance of the four models in the semantic segmentation of the CHAOS dataset.

	ASSD		F1		Precision		Recall	
	Mean±std	p-value	Mean±std	p-value	Mean±std	p-value	Mean±std	p-value
Bayesian ensemble GAN	2.4±0.1	—	97.3±0.1	—	97.6±0.3	—	93.0±0.3	—
Ensemble GAN	2.9±0.2	0.00070	96.1±1.1	0.00017	97.1±0.7	0.00021	90.5±0.5	0.00011
Conditional GAN	12.1±0.6	<0.0001	84.9±2.5	<0.0001	85.5±1.0	<0.0001	69.3±0.9	<0.0001
U-Net	11.02±2.3	<0.0001	83.2±3.7	<0.0001	86.0±1.4	<0.0001	70.5±1.2	<0.0001

Note: The best scores are highlighted in bold.

### 5.2 In-Domain Generalization

Glaucoma is one of the leading reasons of irreversible blindness. Early detection of glaucomatous structural damage has an important impact on treatment. However, the detection of glaucomatous changes is a challenging task in the field of ophthalmology. We evaluated the performance of our Bayesian ensemble GAN and the competitor models on the task of diagnosing glaucoma and quantifying the uncertainty associated with the prediction. Table 3 shows that our Bayesian ensemble GAN method achieved the best classification performance in terms of F1 score and the second-best result in terms of the expected calibration of error. A pairwise t-test on the difference in the F1 scores between the Bayesian ensemble GAN and each competitor model showed that the difference in performance was statistically significant (p<0.001). In general, the results demonstrate that ensemble approaches, such as Masksemble, ensemble GAN, BatchEnsemble, and our Bayesian ensemble GAN model, contribute to a high performance of in-domain generalization for accurate diabetic retinopathy diagnosis.

**Table 3:** Classification performance for glaucoma images.

# member (M)	F1 (%) (↑)				ECE (↓)		
	2	5	10	p-value	2	5	10
Single	84.4			0.00030	0.084		
Ensemble GAN	85.6±0.2	85.8±0.2	86.3±0.3	<0.0001	0.041±0.002	0.078±0.002	0.064±0.003
MC dropout	67.0±0.2	79.3±0.6	81.7±0.5	<0.0001	0.052±0.001	0.055±0.010	0.050±0.018
Masksemble	82.7±0.5	82.0±0.4	81.7±1.1	<0.0001	0.064±0.004	0.049±0.007	0.061±0.012
BatchEnsemble	84.5±0.1	86.5±0.1	87.1±0.2	<0.0001	0.035±0.003	0.071±0.002	0.066±0.002
Bayesian ensemble GAN	86.3±0.1	86.9±0.1	87.8±0.1	—	0.062±0.001	0.068±0.002	0.040±0.001

Note: The best scores are highlighted in bold.

### 5.3 OOD Detection

**Fig. 4:**
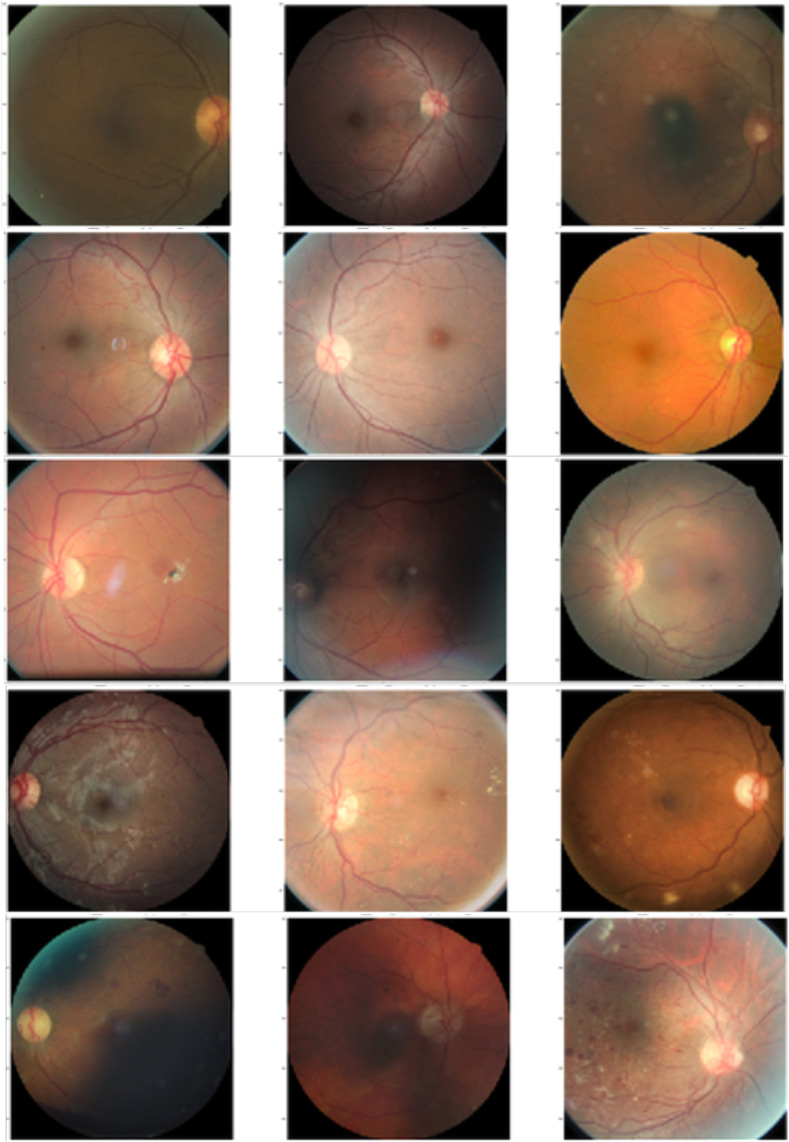
Example images from the glaucoma detection dataset that we used for training.

**Fig. 5:**
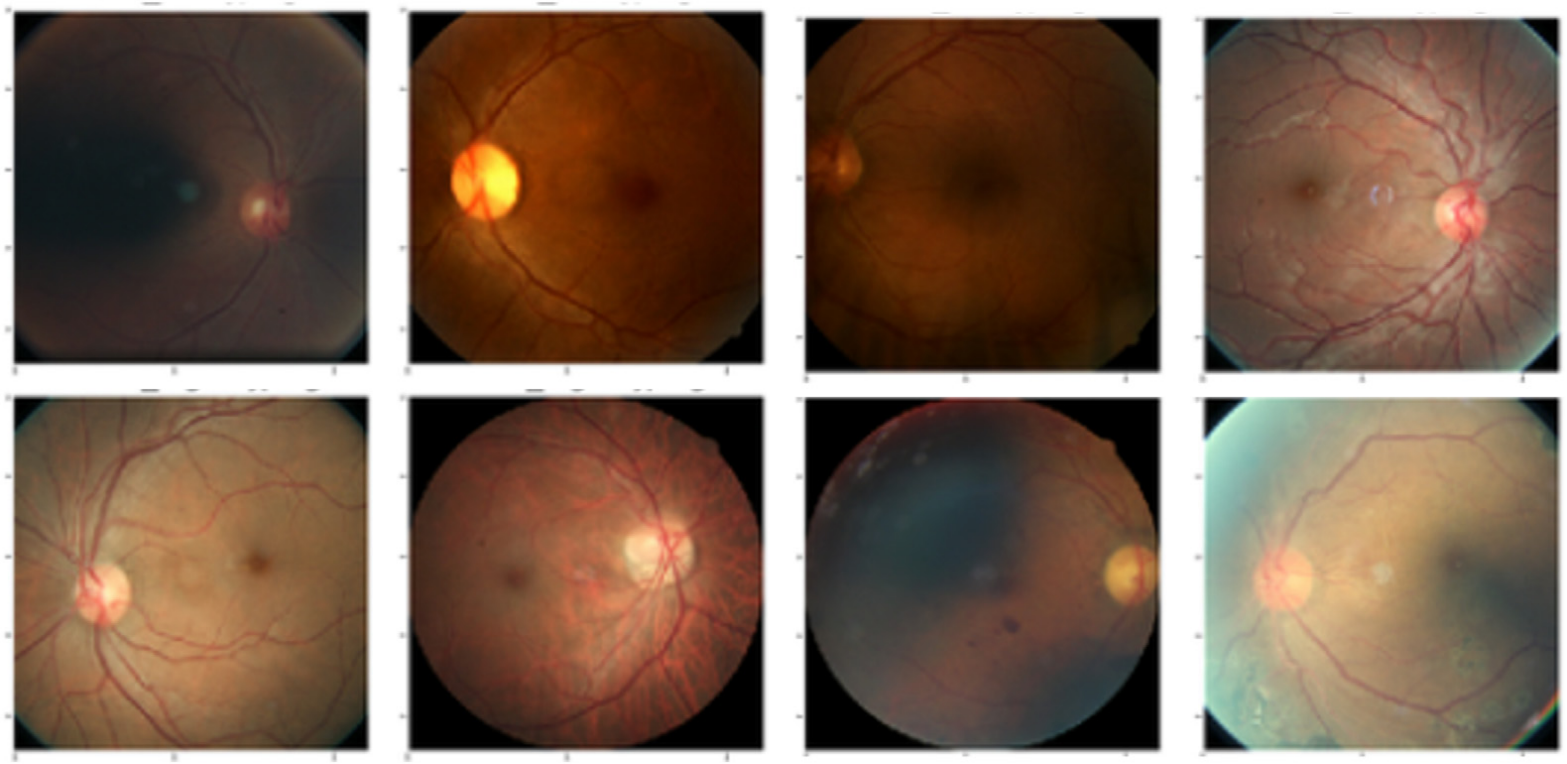
Example images from the REFUGE dataset that we used for OOD detection.

Domain shift often occurs in medical datasets where detecting OOD samples is an important task in clinical diagnosis. Model uncertainty can be used for OOD detection. For this experiment, we regarded the retinal glaucoma detection dataset^34^ (Fig. 4) as the within-distribution samples and performed OOD detection with the REFUGE dataset^35^ (Fig. 5). Table 4 displays the performance metric of area under the receiver operating characteristic (AUROC) scores for the models showing that the Bayesian ensemble GAN outperformed all the comparator models. A pairwise t-test on the difference in the best AUROC scores between the Bayesian ensemble GAN and each comparator model showed that the difference in the performance was statistically significant (p<0.01).

**Table 4:** OOD detection for glaucoma images.

# Member	AUROC (%) (↑)			
	2	5	10	p-value
Single		68.42		0.0071
Ensemble GAN	76.89±0.1	77.98±01	78.06±0.1	<0.0001
MC dropout	68.03±0.3	69.79±0.2	72.22±0.2	<0.0001
Masksemble	71.22±0.5	72.04±1.1	70.95±1.4	<0.0001
BatchEnsemble	74.38±0.1	72.61±0.3	75.04±1.0	<0.0001
Bayesian ensemble GAN	77.02±0.1	77.92±0.2	79.43±0.1	—

Note: The best score is highlighted in bold.

## 6 Discussion

The development and training of machine learning algorithms are often based on the assumption that the frequencies of samples in different classes of the training dataset are similar. However, real-world medical imaging data tend to have long-tailed distributions where many classes are represented by rarely occurring samples. The low number of training samples in such tail classes makes it challenging to learn optimal classification boundaries in the feature space. Existing approaches to address this class-imbalance problem suffer from overfitting or the need for a careful choice of the classifier weights. The deep Bayesian ensemble learning model that we proposed in this paper is based on the observation that rare classes have higher uncertainties and wider confidence intervals in the prediction space than the more frequently occurring classes. By incorporating these uncertainty estimates into the prediction model, we can expand the decision boundaries to the less frequent classes and thus help the classifier to generalize toward the rare and unseen conditions. Using this principle, our method is able to learn the representation of the long-tailed data distribution more efficiently and thus detect the OOD samples more accurately than existing approaches.

We evaluated our method in terms of three imaging tasks. The semantic segmentation task evaluated the ability of our method to perform representation learning of the long-tailed distribution. The in-domain generalization task evaluated the ability of the method to make accurate predictions on the same population from which the training dataset was derived. The OOD detection task evaluated the ability of the method to make accurate predictions on a different population than that from which the training dataset was derived. Our results based on evaluations of four different publicly available challenge datasets show that our proposed deep Bayesian ensemble learning model significantly outperformed the state-of-the-art comparator models across all of the performance metrics in these three tasks.

The high performance of our method can be attributed in part to the use of pretrained discriminators, where the dual output of the generator was passed as both global and local feature vectors to the three individual discriminators. The local features provide detailed information on the edges of the input images, whereas the global features provide high-level information. Moreover, having two adversarial losses for both global and local discriminators, combined with the binary cross-entropy loss of the generative model, resulted in better recognition and smoother segmentation boundaries than those derived from only one adversarial loss.

Although our deep Bayesian ensemble learning model offers several advantages and demonstrates good performance over existing methods, it has some limitations. The model was implemented by use of GANs, the training of which is computationally expensive and time-consuming, especially for large datasets. Also storing multiple models can be memory-intensive, especially if the models are large. These limitations provide topics for future work.

## 7 Conclusions

We introduced a method for the representation learning of the long-tailed distributions and OOD samples in medical imaging data. Based on the observation that rare classes have high uncertainty in the prediction space, we incorporated the uncertainty in identifying reliable examples using Bayesian approximation and ensemble classifiers to assign the class labels based on a consensus of high-confidence predictions. Our experimental results show that the training of the proposed deep Bayesian ensemble learning framework with dynamic MC dropout and a combination of losses yielded a better generalization of the learned classifier to unseen samples in the tasks of semantic segmentation, in-domain generalization, and OOD detection than what was obtained with state-of-the-art comparator models.

## Code, Data, and Materials Availability

The code utilized to derive the results presented in this study is available upon request from the corresponding authors. The datasets used and analyzed in this study are available from the public repositories indicated in Sec. 4.1.
